# Inflammation at the crossroads of *Helicobacter pylori* and COVID-19

**DOI:** 10.2217/fmb-2021-0250

**Published:** 2021-12-17

**Authors:** Ileana Gonzalez, Cristian Lindner, Ivan Schneider, Miguel A Morales, Armando Rojas

**Affiliations:** 1 Biomedical Research Labs, Catholic University of Maule, Talca, Chile; 2 Molecular & Clinical Pharmacology Program, Institute of Biomedical Sciences, University of Chile, Santiago, Chile

**Keywords:** COVID-19, *Helicobacter pylori*, inflammation, SARS-CoV-2

On 11 March 2020, the WHO declared the COVID-19 (SARS-CoV-2) outbreak a pandemic, initially reported in Wuhan, China. Its death rate and global expansion have made it one of the deadliest pandemics in history, with more than 4.8 million confirmed deaths. At present, a compelling body of evidence indicates that the most vulnerable patients to acquire SARS-CoV-2 infection, as well as having an increased risk of hospitalization and death due to severe forms of COVID-19, are those with pre-existing medical conditions or illnesses, particularly noncommunicable diseases (NCDs) [1]. In this context, the interplay between COVID-19 and NCDs is widely supported by a significant number of studies connecting certain pre-existing conditions, such as chronic heart failure, coronary heart disease, hypertension, obesity, chronic lung diseases and diabetes mellitus, to a more severe clinical course of COVID-19. These comorbidities may play a crucial role not only by increasing susceptibility to infection with SARS-CoV-2, but also by contributing to the risk of a more severe course of the disease. From the mechanistic point of view, the central point appears to be the contribution of basal inflammatory states affecting many organs in all of these pathological entities.

However, the COVID-19 outbreak also converges with another burden comprised of many pre-existing infectious diseases like malaria, HIV/AIDS, viral hepatitis and tuberculosis, with higher prevalence rates in particular geographical zones, while others, such as *Helicobacter pylori* infection, are widely spread out and present in more than half of the world's population (4.4 billion people) [2]. In this particular burden of infectious diseases, a body of emerging data suggests that *H. pylori*-infected people may be more susceptible to SARS-CoV-2 infection and severe forms of COVID-19 [3,4]. *H. pylori* is a Gram-negative, microaerophilic and spiral-shaped bacterium, which is now recognized as the etiologic agent of gastritis and peptic ulcers as well as a major risk factor for gastric cancer. *H. pylori* infection occurs at an early age and the course of infection is variable depending not only on the intrinsic pathogenic attributes of the bacteria [5] but also on host factors [6].

*H. pylori* infection is an important initiating and promoting step of gastric carcinogenesis because of its contribution to the setting of a chronic state of inflammation, which may evolve toward atrophy of the glands, intestinal metaplasia (IM) and gastric cancer. According to Correa's cascade [7], noncardia gastric cancer development goes through a series of mucosal changes from nonatrophic gastritis to atrophic gastritis (AG), IM, dysplasia and adenocarcinoma. While *H. pylori* eradication could likely regress AG, the presence of IM may be a point of no return in this sequence of pathological changes. IM is, therefore, a clinically relevant lesion occurring after the setting of chronic inflammation triggered by *H. pylori* infection [8]. In this context, the high burden of IM in *H. pylori*-infected patients is particularly relevant because of the increased expression of SARS-CoV-2 entry receptors ACE2 and TMPRSS2 in the affected gastric mucosa, mainly due to the migration of intestine-specific cell types, including enterocytes, within the gastric lining [9]. Additionally, gastric mucosa with IM usually occurs alongside parietal cell loss and leads to an elevation of intragastric juice pH. Consequently, the SARS-CoV-2 virus does not become inactivated by the low pH of the stomach acid [10]. Furthermore, some evidence suggests that *H. pylori* infection is related to a diverse and wide range of extragastric diseases, which are particularly important in the context of COVID-19, including neurological, dermatological, cardiovascular, metabolic, ocular, allergic, renal and respiratory diseases [11,12].

It is worth noting that the lungs and the cells that constitute the digestive system share a common embryological origin [13], and thus the chronicity of *H. pylori* infection in the gastric epithelium may induce systemic effects and contribute to tissue damage in distant sites from the primary infection organ, such as the lungs. A compelling body of data demonstrates that *H. pylori* can colonize the oral cavity. Thus, the colonization of the upper respiratory system mucosa has been associated with the onset of diseases such as sinusitis, adenotonsillar hypertrophy, pharyngitis and laryngitis. Furthermore, studies have highlighted the role of gastric aspiration in airway inflammation and lung diseases in *H. pylori*-infected patients [14–18]. Of note, airway inflammation may not only occur by the direct effects of *H. pylori* or some of its components, such as toxins, due to tracheobronchial aspiration, but also by the induction of a molecular mimicry mechanism [19]. Orotracheal installation of *H. pylori* in animal models induced morphological changes in the lung tissue with the recruitment of inflammatory cells and sustained production of IL-1β, IL-8 and TNF-α, as well as markers of endothelial dysfunction such as ICAM and V-CAM [20]. In any case, tracheobronchial aspiration may trigger a robust proinflammatory response and then enhance the subsequent SARS-CoV-2-mediated acute lung injury. Lung inflammation is the main cause requiring hospitalization in up to 20% of COVID-19 cases, with life-threatening acute respiratory distress syndrome (ARDS) affecting 75% of COVID-19 patients transferred to intensive care units [21].

Proton pump inhibitors (PPIs) are one of the most consumed drugs among *H. pylori*-infected patients, as part of the eradication therapy and clinical management of some digestive symptoms [22,23]. Recent studies have shown that the use of PPIs not only increased susceptibility to SARS-CoV-2 infection, but also influenced the risk of developing severe clinical outcomes of COVID-19, greater than those who were not receiving PPI therapy [24]. Very recently, some authors have posited that COVID-19 should be regarded as a systemic disease, causing lung-centered injury, mainly due to the disruption of vascular endothelium functions [25–28]. The vascular endothelium is known as a key player in the setting and maintenance of vascular homeostasis by regulating many vascular functions, such as blood fluidity and fibrinolysis, vascular tone, angiogenesis, monocyte/leukocyte adhesion and platelet aggregation [29–31]. A growing body of evidence supports the important roles of endothelial cells at inflammation sites, where they not only become active participants but also actively regulate the proinflammatory and anti-inflammatory responses [32]. Of note, many markers of vascular endothelial dysfunction and altered endothelial cell integrity are related to poor outcomes in SARS-CoV-2 infections [33,34], supporting the suggestion that endothelial biomarkers and functional tests, such as flow-mediated dilation, could be useful for the risk stratification of critically ill patients with COVID-19 [35]. Interestingly, *H. pylori* infection also impaired endothelial function through multiple mechanisms, such as increased reactive oxygen species production, oxidative stress, decreased nitric oxide formation, expression of cytokines and microRNAs, abnormalities of lipid and glucose metabolism and the activation of exosome-mediated pathways [36–40]. *H. pylori* eradication also significantly improved endothelium-dependent vasodilation in both patients and mice with *H. pylori* infection. Therefore, *H. pylori*-infected subjects display a dysfunctional basal endothelial state that could be substantially aggravated when the subject infected with SARS-CoV-2.

The COVID-19 pandemic is an international health emergency and a full understanding of its whole pathophysiology context is mandatory for a complete comprehension of risk factors and management. Therefore, the high worldwide prevalence of chronic *H. pylori* infection in humans and the widespread COVID-19 pandemic may represent an important inflammation-prone cross point between two communicable human diseases that should not be ignored.

## Future perspective

The outbreak of the COVID-19 pandemic is shaking up socioeconomic structures worldwide and causing a global health crisis. Since the very beginning of the outbreak, a compelling body of data has emerged highlighting the contribution of NCDs to having an increased risk of hospitalization and death due to severe forms of COVID-19. However, the burden of many infectious diseases has not been considered with the attention it deserves. The COVID-19 outbreak also overlaps with other infectious diseases such as malaria, schistosomiasis, tuberculosis, viral hepatitis, HIV, dengue and even with other neglected tropical diseases (NTD), which are known to affect billions of people, and in the particular case of *H. pylori*, more than half of the world population is infected. The onset and progression of many infectious diseases develop in a complex pathophysiological context, where basal inflammatory and dysregulated immune reactions, sometimes widely distributed in many organs, may represent an unfavorable niche during the onset of a new infection. Considering the high distribution of *H. pylori* infection, more efforts are necessary to understand the true scope of the pathophysiology of infection, with a focus on gastrointestinal manifestations.

The effects of modern society on the climate, the intrusion of humans on wildlife habitats, global travel and trade, unplanned urbanization and overpopulation, represent important factors contributing to the dissemination of animal-borne diseases, and, therefore, more pandemics are expected. Therefore, the complex ecosystem between highly prevalent human infectious diseases such as *H. pylori* and novel pathogens must not be overlooked.
